# Diagnostic journey in Spinal Muscular Atrophy: Is it still an odyssey?

**DOI:** 10.1371/journal.pone.0230677

**Published:** 2020-03-23

**Authors:** Maria Carmela Pera, Giorgia Coratti, Beatrice Berti, Adele D’Amico, Maria Sframeli, Emilio Albamonte, Roberto de Sanctis, Sonia Messina, Michela Catteruccia, Giorgia Brigati, Laura Antonaci, Simona Lucibello, Claudio Bruno, Valeria A. Sansone, Enrico Bertini, Danilo Tiziano, Marika Pane, Eugenio Mercuri

**Affiliations:** 1 Pediatric Neurology, Università Cattolica del Sacro Cuore, Rome, Italy; 2 Centro Clinico Nemo, Fondazione Policlinico Universitario Agostino Gemelli IRCCS, Rome, Italy; 3 Department of Neurosciences, Unit of Neuromuscular and Neurodegenerative Disorders, IRCCS Bambino Gesù Children's Hospital, Rome, Italy; 4 Department of Neurosciences, and Centro Clinico Nemo Sud, University of Messina, Messina, Italy; 5 Neurorehabilitation Unit, Neuromuscular Omnicentre Clinical Center, Niguarda Hospital, University of Milan, Milan, Italy; 6 Center of Experimental and Translational Myology, IRCCS Istituto Giannina Gaslini, Genoa, Italy; 7 Institute of Genomic Medicine, Università Cattolica del Sacro Cuore Fondazione, Policlinico Universitario Agostino Gemelli IRCCS, Rome, Italy; Providence Care Hospital, CANADA

## Abstract

**Background:**

The advent of new therapies has increased the need to achieve early diagnosis in Spinal Muscular Atrophy (SMA). The aim of the present study was to define the age of diagnosis in the three main types of SMA with pediatric-onset and the timing between the recognition of clinical signs and confirmed genetic diagnosis.

**Methods:**

All patients with a confirmed diagnosis of type I, II, III SMA followed in 5 Italian centers were included in this study, assessing age at symptoms onset, presenting sign or symptom, age at diagnosis, interval between clinical onset and diagnosis and type of medical investigations conducted in order to obtain the diagnosis.

**Results:**

The cohort included 480 patients, 191 affected by SMA type I, 210 by type II and 79 by type III. The mean age at diagnosis was 4.70 months (SD ±2.82) in type I, 15.6 months (SD±5.88) in type II, and 4.34 years (SD±4.01) in type III.

The mean time between symptom onset and diagnosis was 1.94 months (SD±1.84) in type I, 5.28 months (SD±4.68) in type II and 16.8 months (SD±18.72) in type III.

**Conclusions:**

Our results suggest that despite improved care recommendations there is still a marked diagnostic delay, especially in type III. At the time new therapies are becoming available more attention should be devoted to reducing such delay as there is consistent evidence of the benefit of early treatment.

## Introduction

Spinal muscular atrophy (SMA) is a genetic recessive disorder caused by mutations in the survival of motor neuron 1 (*SMN1)* gene on chromosome 5q, leading to motoneuron loss and subsequent muscular atrophy and weakness [1,2]. Classically SMA is subdivided into different types according to maximum motor function achieved, with type I to III being the most frequent forms with pediatric-onset.

In type I the onset is before 6 months of age and the ability to sit independently is not achieved. In type II the onset is between 6 and 18 months; type II children achieve the ability to sit but not to walk independently. Type III patients achieve the ability to walk independently and the onset is after 18 months [3–7] [8–10].

The diagnostic process is thought to be relatively easy because of the combination of typical clinical signs. Generalized weakness, more severe in the legs than in the arms, with a proximal more than distal distribution, associated with no facial weakness and severe generalized hypotonia, absent reflexes and a typical respiratory pattern are strongly suggestive of type I SMA and should direct the clinician to consider performing genetic testing, without the need to perform muscle biopsy or other investigations [11–13]. Similarly, in type II patients the observation of similar motor and respiratory pattern, even if milder, is sufficient to consider diagnosis of SMA and to proceed directly with genetic testing. In type III, the milder signs may be less specific and additional investigations, such as electromyography are often used to confirm the clinical suspicion [14–18]. The genetic testing is simple as the gene is relatively small and approximately 95% of the mutations are represented by deletions in the exons 7 and 8.

Despite the typical clinical features and the ease in performing the genetic analysis, a recent review of the diagnostic process in SMA [19] reports that this is not always so straightforward and that there is often a delay between the onset of clinical signs and confirmed diagnosis in all types of SMA. Achieving a diagnosis is always important, not only for genetic counseling but also to implement disease-specific standards of care [17,20,21]. The recent advent of new therapeutic options, already commercially available, has further increased the need to confirm diagnosis as early as possible as early treatment has been associated with better outcome [22–25].

The aim of the present study was to define the age of diagnosis in the three main types of SMA with pediatric-onset and the timing between recognition of clinical signs and a confirmed genetic diagnosis over the last 2 decades. We were also interested in identifying the most frequent signs that raised the suspicion for SMA and to assess the investigations performed as part of the diagnostic pathway.

## Materials and methods

The study included 5 tertiary Italian Neuromuscular Center involved in the diagnosis and follow- up of SMA patients (2 located in the northern part of Italy, 2 in the center and 1 in the south).

The study was approved by the Ethics Committee of each centers (Fondazione Policlinico Universitario Agostino Gemelli IRCCS, Bambino Gesù Hospital, Gaslini, Nemo Milano, Messina).

Parents of participants and patients were informed that the data collected as part of our routine clinical assessment were going to be used anonymously for an observational study defining the natural history of the diseases and they all gave written consent.

Data from the clinical charts were collected. As regular genetic testing for SMA has been available since 1995 [10,26,27] it was decided to include patients born after 1996 in order to have more consistent results.

All patients with a confirmed genetic diagnosis of SMA (type I, II, III) with mutations in the *SMN1* gene, including deletions, duplications and point mutations, born between January 1996 and July 2019 in whom anamnestic and clinical reports were available were included in this study.

The following information was collected: date of birth, family history of SMA, age at symptoms onset, the person who suspected the diagnosis, presenting sign or symptom, age at diagnosis, the interval between SMA symptom onset and diagnosis, type of medical investigations conducted in order to obtain the diagnosis.

The patients who had a positive family history of SMA were not included in statistical analysis.

“First symptom” was defined as any sign or symptom reported by a physician or a parent/caregiver that was suspicious for SMA. Age at diagnosis was defined as the date of the genetic test.

Descriptive statistic was conducted to analyze the data.

The Mann-Whitney U test was used to compare age at diagnosis between patients born in the decade 1996–2006 and those born later. An additional analysis was used to establish possible differences with children born in the last 7 years. A similar approach was also used to compare the interval between onset of clinical signs and diagnosis in the same subgroups. The level of statistical significance was defined for p-value <0,005.

## Results

We identified 494 children diagnosed with SMA in our records who were seen in our clinic after 1996. Of these, 5 were excluded because not all the appropriate information was available. Nine children (1 type I, 4 type II, 4 type III) were not included in the analysis because diagnosis was received by a positive familial history (e.g. siblings).

The final cohort included 480 patients, 191 affected by SMA type I, 210 by type II and 79 by type III.

### Symptoms onset

#### Type I (n = 191)

The mean age at symptom onset was 2.75 months (range 0–10 months, SD ±1.96). One hundred five patients (54.97%) had symptom onset before 3 months of age, 68 (35.60%) between 3 and 6 months and 18 (9.42%) after 6 months. In 110 children the symptoms were first recognized by parents (62.83%), and in the remaining 71 (37.17%) by a pediatrician/child neurologist.

#### Type II (n = 210)

The mean age at symptom onset was 10 months (range 3–24 months, SD ±3.96). Twelve patients (5.71%) had symptom onset before 6 months of age, 188 (89.52%) between 6 and 18 months and 10 (4.76%) after 18 months. In 157 children the symptoms were first recognized by parents (74.76%), 2 by teachers (0.95%), and in the remaining 51 (24.28%) by a pediatrician/child neurologist.

#### Type III (n = 79)

The mean age at symptom onset was 32 months (SD ±37.92, range 9 months-15 years). None (0%) had symptom onset before 6 months of age, 38 (48.10%) between 6 and 18 months and 39 (49.37%) after 18 months. Of the 38 patients who had symptom onset between 6 and 18 months, only 2 (5.26%) had onset before 12 months, 17 (44.74%) between 12 and 15 months, 19 (50.00%) between 16 and 17 months. Of the 39 patients who had symptom onset after 18 months, 8 (20.51%) had onset before 24 months, 15 (38.46%) before 36 months, 7 (17.94%) before 48 months and 9 (23.08%) after 48 months. In 67 children the symptoms were first recognized by parents (84.81%), the remaining 12 (15.18%) by a pediatrician or other health-related professionals. In 2 children the concern was raised following admission to the ER for unrelated causes (severe headache). Table 1 describes the first symptoms subdivided by SMA type.

**Table 1:** First symptoms identified by parents/caregivers/health care professionals subdivided by SMA type.

SMA I (n:191)			SMA II (n:210)			SMA III (n:80)		
First symptoms identified	N	%	First symptoms identified	N	%	First symptoms identified	N	%
Hypotonia (general)	113	59.16%	Not acquired standing position	83	39.52%	Unsteady ambulation	23	28.75%
Developmental delay (head control)	33	17.28%	Developmental delay (sitting position)	43	20.48%	Frequent falls	18	22.50%
Absence of antigravitary movements	15	7.85%	Hypotonia (lower limbs)	38	18.10%	Difficulty in rise from the floor	10	12.50%
respiratory distress	15	7.85%	Not acquired crawling in time	4	1.90%	Difficulty in stair's climbing	9	11.25%
Developmental regression	7	3.66%	Failure to thrive	1	0.48%	Developmental delay	4	5.00%
Feeding related problems	6	3.14%	Respiratory infections	1	0.48%	Developmental regression	3	3.75%
Absence of deep tendeon reflexes	2	1.05%				Running difficulties	3	3.75%
						‘Clumsy’ movements	3	3.75%
						Muscle Weakness	2	2.50%
						Toe walking	2	2.50%
						Accidental finding	2	2.50%
						Tremor	1	1.25%

### Diagnostic pathway

Table 2 summarize the assessments performed in the SMA patients before performing genetic testing.

**Table 2:** Assessments conducted by SMA patients before performing genetic testing.

EXAM	SMA I (n:191), %	SMA II (n:210), %	SMA III (n:80), %
Only SMN1 molecular analysis	67.02%	56.19%	40%
EMG/ENG	23.04%	45.71%	60%
Brain MRI	5.24%	13.81%	17.5%
Muscle MRI	2.09%	0.00%	1.25%
Metabolic disorders screening	6.81%	1.90%	1.25%
Muscle biopsy	2.62%	2.38%	12.50%
EEG	5.24%	4.29%	1.25%
Brain ultrasound	4.71%	0.95%	0.00%
Other genetic testing	1.05%	0.00%	0.00%

Key to table:

EMG: Electromyography; ENG: Electroneurography; MRI: Magnetic Resonance Imaging; EEG:

Electroencephalography.

### Age at diagnosis

#### Type I (n = 191)

**Fig 1:**
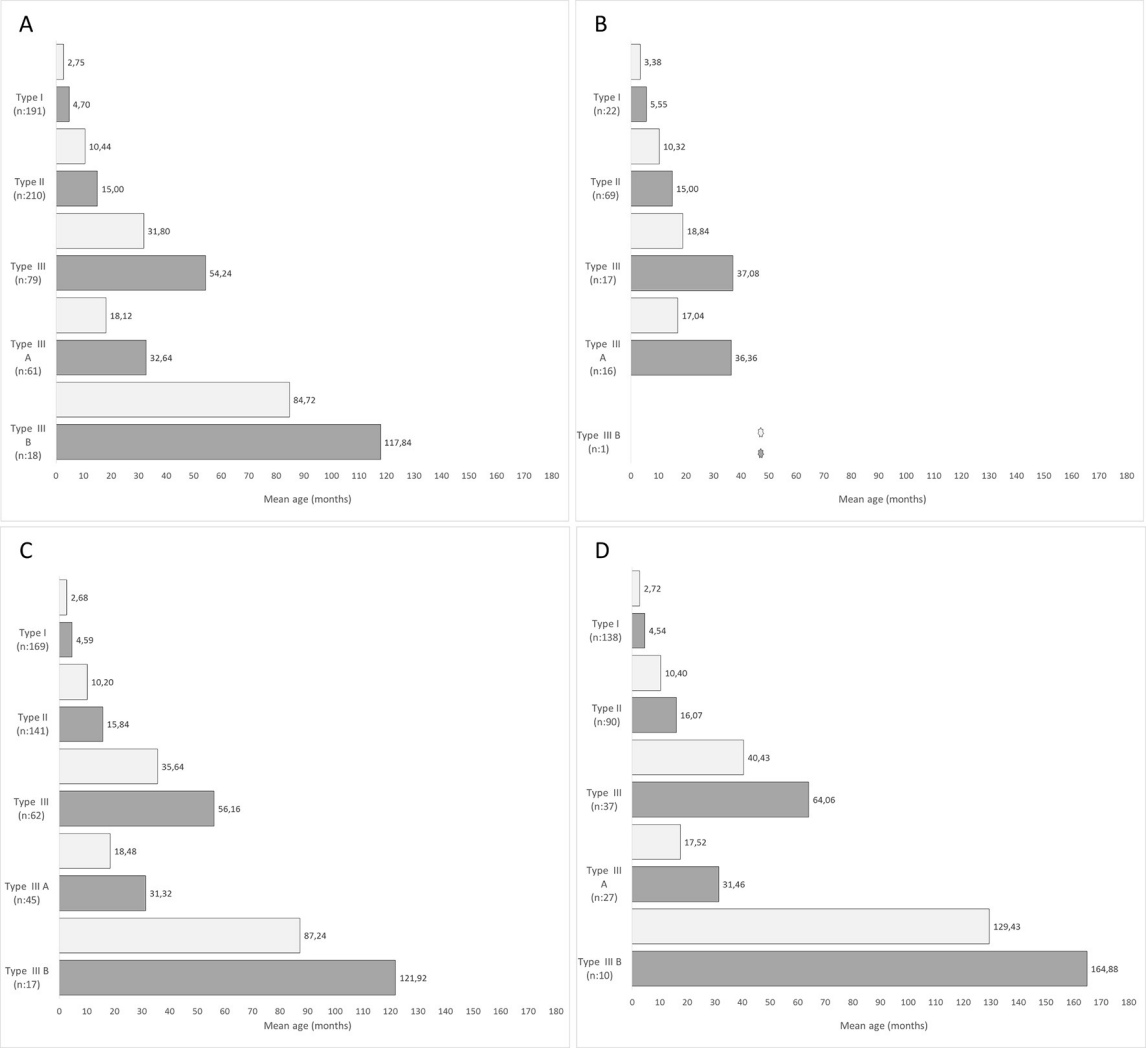
Onset and diagnosis age according to SMA type. Mean age at onset (light grey) and mean age at diagnosis (grey) bar subdivided by SMA type and diagnostic time frame. Panel A: Whole cohort from 1996 to 2019; Panel B: 1^st^ decade, from 1996 to 2006; Panel C: 2^nd^ decade, from 2007 to 2019. Panel D: Last 7 years, from 2012 to 2019. In panel B, only one III b patient was available. Data on his mean age at onset is shown with a light grey star and the mean age at diagnosis with a dark grey star.

The age at diagnosis ranged between 10 days and 13.23 months, mean age: 4,70 months (SD ±2.82). In 63 patients (32.98%) the diagnosis was achieved before 3 months, in 66 (34.55%) between 3 and 6 months and in 62 (32.46%) after 6 months. The mean time between symptom onset and genetic diagnosis was 1.94 months (SD±1.84; range: 0–10.3 months). (Fig 1)

#### Type II (n = 210).

The age at diagnosis ranged between 5 months and 4 years 5 months (mean age: 15.6 months (SD±5.88). In 2 patients (0.95%) the diagnosis was achieved before 6 months, in 140 (66.67%) between 6 and 18 months and in 68 (32.38%) after 18 months. The mean time between symptom onset and genetic diagnosis was 5.28 months (SD±4.68; range: 0–35 months). (Fig 1)

#### Type III (n = 79)

The age at diagnosis ranged between 10 months and 18 years (mean 4.34 years; SD±4.01). In none (0%) the diagnosis was achieved before 6 months, in 4 (5.06%) between 6 and 18 months and in 75 (94.94%) after 18 months. The mean time between symptom onset and genetic diagnosis was 16.8 months (SD±18.72; range: 0–102 months). (Fig 1)

Table 3 summarizes statistical analysis performed between age at diagnosis, interval between onset of clinical signs and diagnosis.

**Table 3:** Significance levels between age at diagnosis, onset of clinical signs and diagnosis.

	SMA I		SMA II		SMA III	
Time interval	Age at diagnosis in months, *Mean (SD)*	Interval between onset of clinical signs and diagnosis in months *Mean (SD)*	Age at diagnosis in months, *Mean (SD)*	Interval between onset of clinical signs and diagnosis in months *Mean (SD)*	Age at diagnosis in months, *Mean (SD)*	Interval between onset of clinical signs and diagnosis in months *Mean (SD)*
1996–2006	5,55 (2,93)	2,17 (1,92)	15 (5,76)	4,68 (4,2)	37,08 (18,72)	24,24 (18,12)
2007–2019	4,95 (2,80)	4,60 (1,83)	15,84 (5,88)	5,52 (4,92)	56,16 (52,8)	17,4 (64,32)
*p-value*	*0*,*115*	*0*,*698*	*0*,*219*	*0*,*085*	*0*,*504*	*0*,*077*
2012–2019	4,53 (2,87)	1,81 (0,16)	10,44 (3,36)	5,64 (5,04)	62,04 (60,96)	10,68 (78)
*p-value*	*0*,*092*	*0*,*469*	*0*,*624*	*0*,*079*	*0*,*619*	*0*,*015*

## Discussion

The analysis of the onset of clinical signs in our large cohort of SMA patients confirmed previous reports [3,28,29] that in the great majority of type I patients clinical signs are generally identified before 6 months of age, and less than 10% were identified after 6 months. The patients with relatively later-onset had all achieved head control and can be classified as 1.9 according to the Dubowitz decimal classification [3]. These findings expand recent observations obtained in a smaller cohort reporting that at the mildest end of the spectrum in type I the onset may occur after the age of 6 months^29^.

As expected [2], the great majority of type II patients had a clinical onset between 6 and 18 months with approximately 5% of outliers at each end of the spectrum. In contrast, the number of type III patients with onset of clinical signs after 18 months was approximately 50% of all type III. In the patients in whom the signs were identified before 18 months, this generally occurred between 13 and 18 months, after independent ambulation had been achieved. The main concern reported by the parents/caregiver/HCPs was persistent unsteady gate or frequent falls a few months after ambulation had been acquired. Although these results confirm that most patients will fall within the criteria used for the SMA classification [2,3], this is less true for the type III with earlier onset (IIIA) and the possibility to have outliers is present in all types.

A recent review [19] reported a diagnostic delay in SMA, measuring the mean interval between the age at clinical onset and the age at genetic diagnosis based on a number of studies published before 2015 in which these data were available. The review showed a progressive increase in the interval from type I to type III.

The results were not easily comparable because of the different design of the studies but some differences could be noted. In our type I patients the mean interval between clinical onset and diagnosis was 1.94 months. This value is lower than the mean value reported in the review.

We also found that, despite having a similar age at clinical onset, in our type II patients the mean interval was also much shorter than in the type II patients in the published review.

The results in the type III cohort cannot be easily compared as this is a more heterogeneous group and the results may be dependent on the percentage of patients with earlier or later onset and this information was not available in the previous review paper. Our type III cohort was subdivided into IIIa and IIIb according to whether the diagnosis was made before or after the age of 3 years. This was helpful to highlight that the diagnostic interval between IIIa and IIIb is much shorter. In the IIIa patients, parents were often concerned about persistently unstable gait or ‘clumsiness’ a few months after ambulation had been achieved and this prompted further investigations. In older patients in whom the clinical signs became obvious after the age of 3 years, the signs were often milder and less specific, and parents reported a ‘wait and see’ attitude, a longer time before referral to a specialist, that justifies the longer interval before diagnosis.

Interestingly although type III had more delay than the other groups, this was the only clinical group in which a significant improvement was noted in recent years compared to the previous decade.

In the other SMA types, the interval between clinical onset and diagnosis in the overall cohort was not significantly different between the two decades explored or between the first decade and the last 7 years. The choice of selecting the last 7 years, from 2012, was related to a possible increased awareness and education in the last few years, following the implementation of the standard of care recommendations published in 2007 and the advent of active clinical trials. While the time to perform the genetic test, from blood taken to results is likely to have improved in the last few years, reflecting a wider availability and improvement of the diagnostic services, the overall time between onset of clinical signs and genetic diagnosis did not change. This was particularly true for the patients in whom the diagnostic workout was initially performed in local hospitals. Although our study includes only tertiary care centers, a proportion of children were referred to us only after diagnosis. In these patients, a diagnosis of SMA was often considered only after an extensive number of tests ruling out other diseases had been performed and the delay may also reflect the time it takes to get a referral to and appointment with a specialist.

As already reported in the previous review the interval increased from type I to type III. A possible explanation is that with decreasing severity, the clinical signs are milder and can be less specific, as also proven by the highest number of investigations such as brain MRI and EMG, performed more often in type III, and progressively less in type II and type I. Similarly, muscle biopsy, that used to be one of the key diagnostic elements before the gene was identified and is not part of the current recommendations for diagnosis and care [19], was more often performed in type III than in the other 2 types.

The only tests that were performed more regularly in type I than in the milder forms were metabolic tests and other genetic tests, mainly tests for Prader Willi, Pompe disease and Myotonic Dystrophy, that in some centers are routinely performed in weak floppy infants.

## Conclusions

In conclusion, our results showed that age of onset of clinical signs can identify the great majority of type I and type II cases, but this is not always true for type III, as clinical signs were often identified after ambulation was achieved but before the age of 18 months.

Our results also showed overall shorter diagnostic intervals compared to previous studies. The interval was shorter in type I, this probably reflects the wider availability of genetic tests even in peripheral hospitals but also partly as the result of increased awareness of the disease in the last few years following the advent of new therapies. These findings should be interpreted with caution as they reflect the experience in our country, and they may not be necessarily extrapolated to countries where healthcare systems are different.

The advent of new therapies and the promising results obtained in presymptomatic patients [30] are paving the way to the possibility of newborn screening that is already in place in a few countries and is likely to have improved time to diagnosis in the future.
